# Hedgehog signaling patterns the outgrowth of unpaired skeletal appendages in zebrafish

**DOI:** 10.1186/1471-213X-7-75

**Published:** 2007-06-27

**Authors:** Yavor Hadzhiev, Zsolt Lele, Simone Schindler, Stephen W Wilson, Per Ahlberg, Uwe Strähle, Ferenc Müller

**Affiliations:** 1 Institute of Toxicology and Genetics, Forschungszentrum Karlsruhe, D-76021 Karlsruhe, Germany; 2 Department of Anatomy and Developmental Biology, University College London, Gower street, London, WC1E 6BT, UK; 3 Department of Evolutionary Organismal Biology, Evolutionary Biology Centre, Uppsala University, SE-752 36, Uppsala, Sweden; 4 Department of Gene Technology and Developmental Neurobiology, Institute for Experimental Medicine, Szigony u. 43., H-1083, Budapest, Hungary

## Abstract

**Background:**

Little is known about the control of the development of vertebrate unpaired appendages such as the caudal fin, one of the key morphological specializations of fishes. Recent analysis of lamprey and dogshark median fins suggests the co-option of some molecular mechanisms between paired and median in Chondrichthyes. However, the extent to which the molecular mechanisms patterning paired and median fins are shared remains unknown.

**Results:**

Here we provide molecular description of the initial ontogeny of the median fins in zebrafish and present several independent lines of evidence that Sonic hedgehog signaling emanating from the embryonic midline is essential for establishment and outgrowth of the caudal fin primordium. However, gene expression analysis shows that the primordium of the adult caudal fin does not harbor a Sonic hedgehog-expressing domain equivalent to the Shh secreting zone of polarizing activity (ZPA) of paired appendages.

**Conclusion:**

Our results suggest that Hedgehog proteins can regulate skeletal appendage outgrowth independent of a ZPA and demonstrates an unexpected mechanism for mediating Shh signals in a median fin primordium. The median fins evolved before paired fins in early craniates, thus the patterning of the median fins may be an ancestral mechanism that controls the outgrowth of skeletogenic appendages in vertebrates.

## Background

Living primitive chordates like *Branchiostoma *and the agnathan hagfishes have a very simple, non-differentiated caudal fin fringe, whereas lampreys are characterized by separate and differentiated caudal and dorsal median fins [1]. Recent analyses concur that the fossil jawless vertebrates ("ostracoderms"), which have differentiated median fins and either no or one pair (pectoral) fins, form the stem group of the Gnathostomata. This phylogenetic pattern implies that median fins appeared before paired fins [2]. The mechanisms patterning median fins may be ancestral to those used by the paired fins and limbs [3]. Gnathostomes primitively have paired pectoral and pelvic fins, as well as unpaired caudal, anal and at least one dorsal fin, which together is the ancestral morphology well matched by zebrafish [1].

The development of paired appendages such as wings, fins and limbs has been intensively studied and many details about the underlying molecular mechanisms are known (reviewed in [4]). For instance, the signaling molecule Sonic hedgehog (Shh) is a morphogen [5] that emanates from the zone of polarizing activity (ZPA), a domain of cells positioned at the base of the outgrowing paired fin and limb buds [6-8]. Shh expressing cells can mimic the activity of the ZPA and can lead to mirror image duplications of appendages. Thus, Shh is the factor responsible for the patterning activity of the ZPA (reviewed in [9]). Recently, it has been established that the main function of Shh in the ZPA is to counteract the repressing activity of Gli3 thus demonstrating an antagonistic hierarchy in establishing antero-posterior patterning of limbs [10-12].

In contrast to the mechanisms underlying the development of paired appendages, very little is known about the formation of median fins [3,13]. In teleosts, the median fins develop from the embryonic fin folds [3,14] by expansion of the mesenchyme underlying the ectodermal layer of the embryonic fin fold [14]. It has been suggested that the processes of embryonic fin fold and adult median fin formation involve independent genetic mechanisms [3,13]. The fin fold mesenchyme is the source of both the endoskeletal and exoskeletal structures of the adult caudal fin and has been suggested to receive contribution from trunk neural crest [15,16]. Recently, the origin of median fin primordial cells was investigated in dogshark and lampreys and the authors concluded that median fin cells are primarily of somitic origin with some contribution from neural crest [17]. Furthermore, regionalization of *Hox *and *Tbx18 *gene expression in median fin buds was demonstrated in the same study and thus indicated that common molecular mechanisms are utilized by median and paired fins. For example, *hoxb8a *was implicated as an important factor in the outgrowth of the caudal fin of medaka (Oryzias latipes) [18].

The lack of appropriate marker genes to follow the initial development and patterning of median fin primordium together with the late, post-embryonic ontogeny of median fins in Osteichthyes [19] prevents genetic analysis in mutants affecting paired fin development in zebrafish because they are mostly embryonic lethal [8] and die before tail fin morphology can be studied. In particular, a problem which has not yet been possible to address due to the early lethal nature of Hh pathway mutants in zebrafish is whether Hh signaling would have a role in patterning the median fin primordium.

In this report we describe a molecular marker for the earliest phase of adult caudal fin primordium development in zebrafish, which facilitates the detection of median fin precursor cells as early as 1.5 days post fertilization. We provide several lines of evidence, which indicate a crucial role for Hh signaling in the patterning of the caudal fin endoskeletal primordium without detecting a sonic hedgehog secreting zone of polarizing activity.

## Results

**Figure 1:**
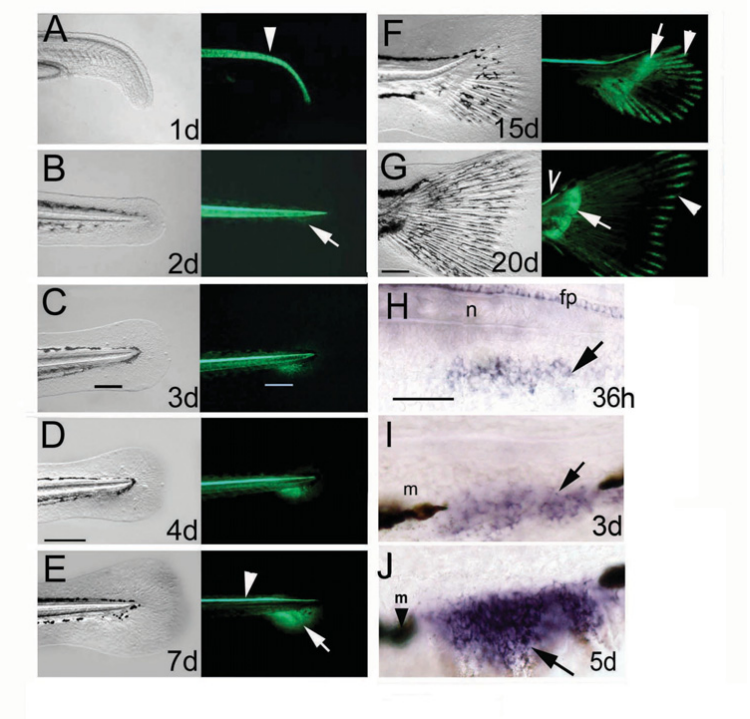
**The ontogeny of the caudal and pectoral fins is marked by continuous GFP expression in the transgenic zebrafish line *2.2shh:gfp:ABC#15***. A-G, The first 20 days of zebrafish caudal fin development. Bright field view of caudal fin on left panels, right panels show fluorescence signals of GFP activity. A, GFP in the notochord is shown by an arrowhead. B, GFP protein is detected from 2 days onwards in the embryonic fin fold mesenchyme. C, D, GFP expression indicates that the caudal fin mesenchyme occupies the gap of the melanophore streak at 3–4 dpf (black and white bars in C). E, GFP in the caudal fin primordium is split anteroposteriorly at 7 dpf (arrow at boundary of domains). Notochord expression is diminished, while floor plate expression remains active (arrowhead). F, G: GFP expression extends caudally and tilts dorsally indicating the formation of adult caudal fin morphology with dorsal and ventral lobes. In late larval development proximal GFP expression is present in the endoskeletal territory (arrows in F, G) and distally in the fin rays (arrowheads in F, G). GFP is expressed continually in the floor plate (open arrowhead in G). H-J, *gfp *mRNA expression in the ACFP is first detected at 1.5 dpf by in situ hybridization (H) then extends ventrally (arrows in I, J) between the melanophores (arrowhead with m). Age of larvae developing at 28°C is indicated in days post fertilization (d). Scale bar in D represents 200 μm (for panels A-F) and 100 μm in G. Scale bar in H represents 100 μm (H-J). Abbreviations; fp, floor plate, n, notochord, m, melanophore.

**Figure 2:**
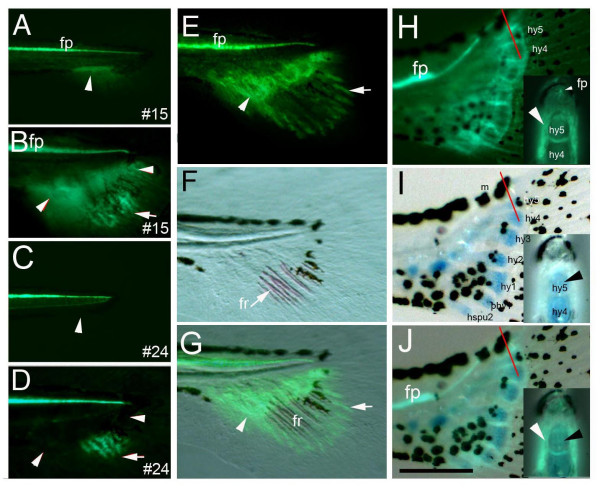
**The proximal GFP domain in the ACFP is independent of the fin rays and marks endoskeletal structures**. Proximal GFP labeling the ACFP and its derivates (arrowheads in A, B) is only observed in one transgenic line (#15) out of 29 lines produced (compare areas with arrowheads in A to C and B to D), while GFP activity in the distal fin rays is present in several transgenic lines containing the *shh *regulatory elements (arrows in B, D) mimicking endogenous *shh *expression of the fin ray tips [21]. E-G, Proximal GFP in ACFP (arrowhead) and distal GFP domains in the fin ray tips (arrow) are physically separated during ossificiation of fin rays. Fluorescent view (E), bright field view (F) and overlay of E and F (G) of caudal fins of 12 dpf larva of transgenic line #15. F, ossification of fin rays (fr, arrow) is detected by alizarin red staining. H-J, GFP is detected in the perichondrium (arrowhead) around, but not in the endoskeletal cartilage of the hypurals marked by alcian blue staining (arrowhead). H, fluorescent view, I, bright field view, J, overlay of H and I of 14 dpf larva (5.5 mm notochord length). Red bars indicate plane of cross sections inserted in H-J. Scale bar in I indicates 120 μm (H-J). Abbreviations: fp, floor plate, n, notochord, hspu, hemal spine, phy, parhypural, hy, hypural, fr, fin ray, m, melanophore. Anatomical structures were identified as described in [19].

To address the role of key signaling mechanisms in median fin patterning appropriate marker genes are required for labeling the median fin primordia. In the lack of marker genes for the earliest events in median fin formation we have exploited a GFP transgenic zebrafish line. As part of a study of floor plate and notochord-specific regulatory elements of *shh *we have generated one stable transgenic zebrafish line that expresses *green fluorescent protein (gfp) *ectopically in the primordia of the median fins from a very early stage of development (Fig. 1) as a result of a position of integration effect (see Fig. 2A–D). The expression of this transgene reporter marks a small group of cells in the embryonic fin fold mesenchyme ventral to the caudal end of the notochord as early as 36 hours post fertilization (hpf) (Fig. 1B, H). The GFP activity gradually expands in a radial manner, generating an expression domain, which strikingly overlaps with the gap in the melanophore streak appearing in the fin fold mesenchyme at 72 hpf (Fig. 1C, I, J). By 7 days post fertilization (dpf), the GFP domain develops into a fan-shape and a new symmetry plane emerges, which splits it into two distinct anterior and posterior regions (Fig. 1E, also see time lapse animation in Additional file Fig. S1). These expression domains correspond to the future dorsal and ventral lobes of the caudal fin after the subsequent bending of the body axis dorsally (Fig. 1F, G and see time lapse movie in Supplementary Fig. S1) and thus represent a molecular correlate of one of the key morphological specializations of the teleosts [20]. The GFP expression further subdivides at around 9 dpf resulting in a stronger proximal and a late developing weaker distal activity (Fig. 1F, G arrows and arrowheads, respectively). The transgene expression in the proximal domain of the median fin primordia is the consequence of an aberrant activation due to a position effect at the integration site and not due to *shh *regulatory elements present in the transgene construct (Fig. 2A–D). The proximal transgene expression domain remains active throughout the subsequent ontogeny of the caudal fin and marks the perichondrium (Fig. 2H–J, Suppl. Fig. S1). These results indicate that the proximal GFP expression domain marks the cells of the adult caudal fin primordium (ACFP) from a very early stage when no obvious cellular features of the ACFP can yet be distinguished and provides a lineage tracer for the formation of endoskeletal structures of the caudal fin.

The distal GFP domain is restricted to the growing tip of the fin rays from 10 dpf onwards (Fig. 1G, Suppl. Fig S1). Importantly, the distal domain of GFP is not due to the position effect, but an inherent property of the transgene construct (Fig 2A–D). This GFP domain is physically separated from the proximal domain marking the ACFP during the process of ossification of the fin rays (Fig. 2E–G) and is consistent with the activation of *shh *expression in the growing tip of fin rays first detected at late larval stages with a suggested function in caudal fin regeneration [21]. Similarly, all other median fins, such as the dorsal and anal fin primordia are marked by both GFP domains during development (see Additional file Fig. S2). The expression of GFP is also present in the paired pectoral fins from 36 hpf in the fin bud mesenchyme disc at 5 dpf and throughout subsequent pectoral fin development (see Additional file Fig. S3). Together, these results indicate that the proximal GFP expression in this transgenic fish is the earliest expressed molecular marker in median fin primordia and a general marker for endoskeletal structures of skeletal appendages in zebrafish. Therefore, the fortuitous expression of GFP in the ACFP in this transgenic line can be exploited to address questions about the patterning of median fin primordia in a similar fashion to the application of enhancer trap and position effect lines marking specific tissues [22,23]. Moreover, the GFP marker in the embryonic fin fold thus facilitates the genetic analysis of ACFP patterning and development in embryonic lethal mutants before these mutants die.

**Figure 3:**
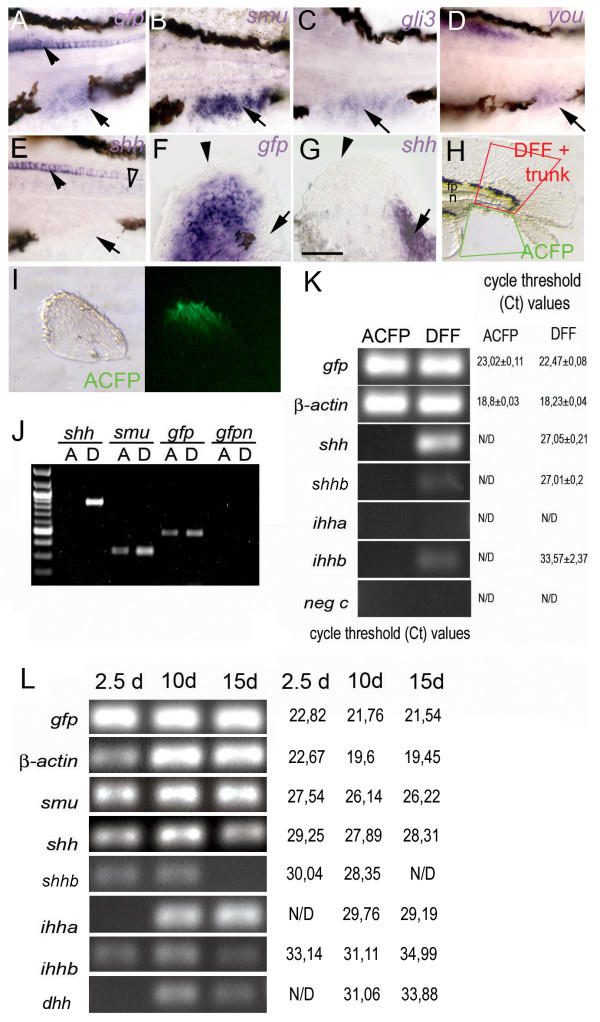
**Hh signaling components but not *shh *are expressed in adult caudal fin primordium**. Expression of *gfp *in caudal fin primordium at 3 dpf (A, arrow) overlaps with *smu *(B, arrow) *gli3 *(C, arrow) and *you *expression (D arrow). E, No *shh *expression is detected in the fin fold mesenchyme (arrow) while a weak residual activity in notochord is detected (open arrowhead). Expression of *gfp *and *shh *overlap in the floor plate (compare A to E, arrowheads). F-G, Expression of *gfp *is lacking in the posterior pectoral fin (arrow, F) where *shh *is expressed (arrow in G). Tail regions of 72 hpf embryos are shown anterior to the left in A-E and isolated pectoral fin buds of 60 h embryos are shown distal to the top posterior to the left in F, G. Scale bar in F represents 100 μm (A-B), 80 μm (C-E) and 40 μm (F, G). H, I, microsurgical preparation of fin fold tissues from 3 dpf zebrafish larvae for RT PCR analysis. H, ACFP tissue was cut by scalpel as indicated by green rectangular area (ACFP). Dorsal fin fold tissue containing notochord and neural tube with floor plate was excised similarly (red rectangular area, DFF). I, ACFP tissue after excision. Right panels show bright field view, left panels are fluorescent views of caudal fin tissues. J, RT PCR analysis of gene expression in the ACFP and DFF tissue samples. *Gfp *and *smu *but not *shh *are expressed in the ACFP. K, Real time PCR analysis of *hedgehog *genes in the ACFP and DFF. No known *hedgehog *genes are expressed in the ACFP. Agarose gel electrophoresis of PCR products in 40 cycles is shown on the left. Ct values of real time PCR cycles are shown on the right. L, Expression of *hedgehog *genes in the caudal fin of zebrafish during development. Agarose gel electrophoresis of real time PCR after 40 cycles on whole caudal fin fold and caudal fin samples are shown. Abbreviations: A, ACFP, D, DFF, d, days post fertilization, gfpn and neg c: gfp negative controls, ND, no specific product detected.

**Figure 4:**
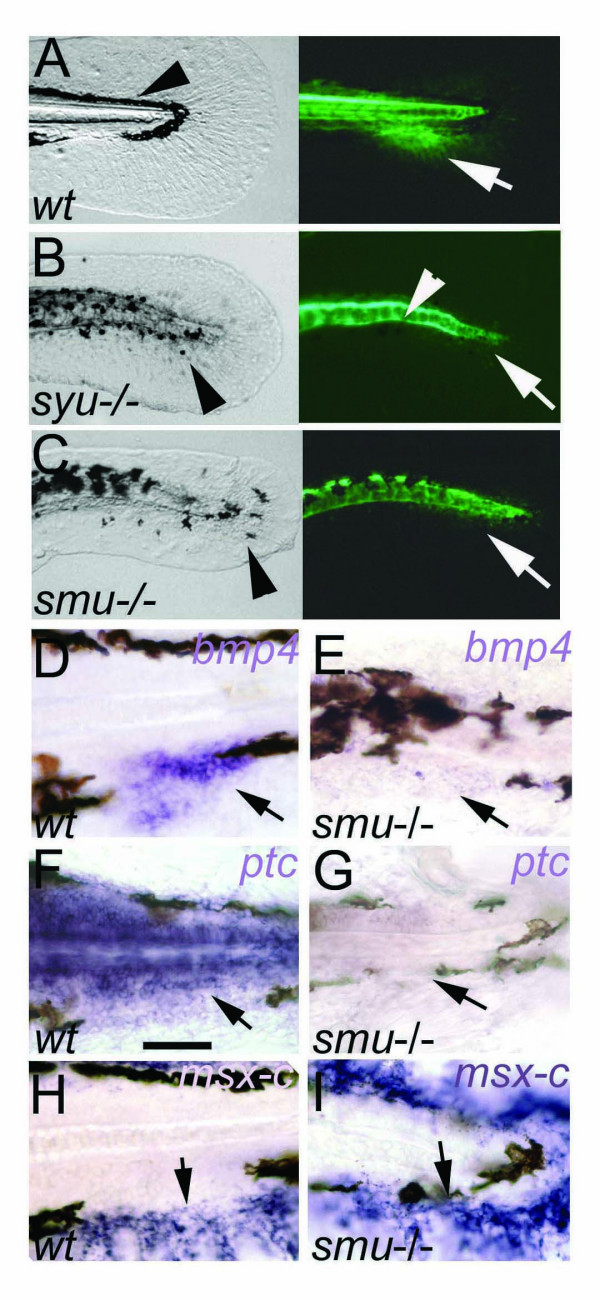
**Hedgehog signaling is required for the patterning of the caudal fin. **A-C: caudal fin primordium development is blocked in *syu*^-/- ^and *smu*^-/- ^mutants. A, Wild type embryo tail showing normal embryonic fin fold and melanophore streak (arrowhead) in bright field (left) and GFP fluorescence (right) marking the adult caudal fin primordium (arrow). B, C, In *syu*^-/- ^and *smu*^-/- ^mutants *gfp *expression in the fin fold is absent (arrows). The melanophores are abnormally arranged (arrowheads in left). Expression of GFP in the floor plate and residual activity in the notochord is present (arrowhead in B, right). D-I, Hedgehog signaling regulates gene expression in the caudal fin primoridum. Expression of *bmp4 *(arrows in D, E) and *ptc *(arrows in F, G) are lost or strongly reduced in *smu*^-/-^. In contrast, the mesenchyme marker *msx-C *is expressed in the embryonic fin fold in wild type (H, arrow) and in *smu*^-/- ^mutants (I, arrow). Lateral views on embryonic tail region of 72 hpf embryos are shown. Scale bar in F indicates 100 μm.

Given the role of Hh signaling from the localized expression of *shh *in the ZPA of paired fins and limbs, we asked whether Hh pathway components are expressed in the ACFP which would indicate the possible existence of a comparable organizing center of the caudal fin/unpaired fins. Interestingly, expression of *smoothened (smu) *was detected specifically in the ACFP (compare Fig. 3A, B, J). *Gli3 *[24] which together with *shh *regulates antero-posterior patterning in the tetrapod limbs [10,12] is also expressed specifically in the ACFP (Fig. 3C). Moreover, the recently described *you *gene which has been implicated as a permissive mediator of Hedgehog signaling [25,26] is also specifically detectable in the ACFP region (Fig. 3D). Taken together, the specific activity of Hedgehog signaling pathway components, such as *smu*, *gli3*, *you *together with the expression of *ptc *in the caudal fin primordium (Fig. 4F) strongly argue for a direct role of Hh signaling in the ACFP.

In contrast, no *shh *or any other known zebrafish *hedgehog *gene expression was detected where the ACFP marker *gfp *expression is clearly detectable at 36, 48 and 72 hpf by whole mount in situ hybridization (Fig. 3A, D and data not shown). To exclude the possibility that the lack of detection of expression of *shh *and other *hedgehogs *in the ACFP is due to low levels below the sensitivity threshold of the whole mount in situ hybridization technique, we have addressed this question by a real time RT PCR approach on microsurgically prepared ACFP and control fin fold samples. ACFP tissue samples were prepared from 3 days old transgenic zebrafish larvae without midline tissues. As control, similar sized dorsal fin fold tissue which also includes *shh *expressing cells such as the floor plate and the notochord (DFF) were excised (see Fig. 3H, I). Expression of GFP was detected in both ACFP and DFF as expected. In contrast, none of the known zebrafish *hedgehog *genes including *shh*, *shhb *(previously named as *twhh*), *ihhb*, [6,27], *ihha *and *dhh *[6,28] were detectable in the ACFP (Fig. 3K, and see Additional file Fig. S4), while *shh*, *shhb *and *ihhb *were detectable in the control tissue sample (DFF) which included the notochord and floor plate, known sources of *shh*, *shhb *and *ihhb*. *Ihha *was described to be active in the chondrocytes of the forming hypurals of the caudal fin in late stages (10 mm larvae, approx. 15 dpf) [28]. We were able to confirm the presence of *ihha *in late stages of caudal fin development (10–15 dpf) by using real time RT PCR (Fig. 3L). In contrast, we have found no evidence for the activity of either *ihh *homologs in the ACFP at 2.5 dpf and 3 dpf using a sensitive real time RT PCR approach (Fig. 3J, Fig. S4). Taken together, none of the known *hedgehog *genes in zebrafish show detectable activity in the ACFP by two independent detection techniques. Importantly, the lack of *shh *or *shhb *expression in the ACFP suggests that the caudal fin primordium does not contain an Hh-expressing domain equivalent to the ZPA of paired appendages. In addition, the GFP transgene is present in the pectoral fin but it is not active in the domain of expression of endogenous *shh *in the ZPA (Fig. 3F, G). These result together with the observation that *shh *is not expressed in the ACFP further support our conclusion that the GFP activity in the ACFP results from a transgenic position effect and does not reflect the tissue specificity of the regulatory elements of the transgene promoter. However, the activity of downstream Hedgehog pathway components suggests a long distance Hedgehog signaling function in the ACFP.

Since Hedgehog pathway components were found to be active in the ACFP we asked whether Hh signaling is required for the formation of the ACFP. To this end, we utilized the GFP transgene expression as marker for the ACFP in *sonic you *(*syu*^-/-^) and *slow muscle omitted *mutants (*smu*^-/-^) that are defective in the function of Shh and the Hh co-receptor Smoothened, respectively [29,30]. GFP expression was strongly reduced or completely lost in the region of the ACFP of the two mutants, indicating that Shh signaling is required for normal patterning of the ACFP (compare Fig. 4A to 4B, C). Similarly, *bmp4 *[31] a marker of median fin bud in dogfish [32] and the Shh target gene *patched *(*ptc*) [33] are expressed in the wild type ACFP but are vastly impaired in Hh pathway mutants at 72 hpf (Fig. 4D, E and 4F, G respectively). Expression of the mesenchyme marker *msx-C *[34] however, was unaffected (Fig. 4H, I) indicating that the ACFP phenotype in *smu*^-/- ^is not due to general loss of fin fold mesenchyme. A further aspect of both *smu*^-/- ^and *syu*^-/- ^mutant phenotypes is the mismigration of melanophores resulting in the loss of the melanophore gap that marks the position of the ACFP in wild type embryos (compare Fig. 3A to 3B, C). The mutant data together with the expression of Shh pathway components clearly indicates that GFP expression in the ACFP requires *sonic hedgehog *function and the activity of the canonical Hh signaling pathway.

**Figure 5:**
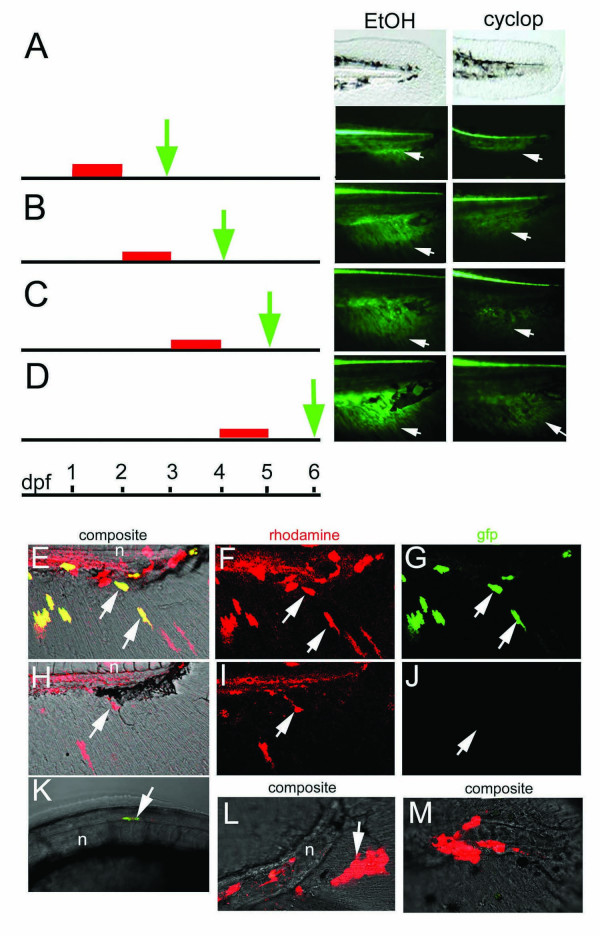
**Continual and direct requirement for Hedgehog signaling in caudal fin development**. A-D, Schematic representation of the time span of the experiment (black stripe), cyclopamine treatment (red stripe) and time of analysis of embryos (green arrow) are shown on the left. Tail of zebrafish embryos at the time of analysis are shown on the right. Bright field (top panels in A) and fluorescence signals (lower panel in A and B-D) of representative samples of zebrafish embryos are shown. A, No GFP expression in the fin fold mesenchyme in cyclopamine treated embryos (cyclop, arrow) in comparison to control ethanol treated embryos (EtOH, arrow). B-D: Cyclopamine treatment from 24 to 48 h results in reduction of GFP expression. E-M: Cell autonomous and non-autonomous requirement for *smu *in development of caudal fin primordium. E-G: transplantation of wild type transgenic cells targeted in the ACFP results in donor cells in the fin fold mesenchyme (arrows in F) with activated GFP (arrows in E, G). H-J: Transplantation of *smu*^-/- ^transgenic cells into wild type embryos results in rhodamine labeled cells in the fin fold mesenchyme (arrow in I) but these cells do not express GFP (arrows in H, J). K: *smu*^-/- ^transgenic cells in the floor plate activate *gfp *expression (arrow). L, M: Transplantation of wt transgenic cells into *smu*^-/- ^non-transgenic embryos results in rhodamine labeled cells excluded from the fin fold mesenchyme (arrows). Tail region of 72 hpf embryos are shown anterior to the left. Abbreviation: n, notochord.

Next, we addressed the question when Hh signaling is required for development of the ACFP. Hh signaling was blocked in a temporally controlled manner by administering the alkaloid drug cyclopamine, a specific inhibitor of Smoothened protein function [35], at different developmental stages. Cyclopamine treatment at 24 hpf to 48 hpf results in loss of transgene expression in the ACFP phenocopying the effects seen in *syu*^-/- ^and *smu*^-/- ^mutants (Fig. 5A to Fig. 4B, C and see Fig. S5). Thus, cyclopamine treatment appears as efficient as blocking the Hh pathway genetically and resulted in eventual lethality of the larvae. When administered at 48, 72 or 96 hpf for 24 hours each time, cyclopamine treatment still resulted in a reduction of the GFP expression domain while controls were unaffected (Fig. 5B–D and see Additional file Fig. S5). The effect of cyclopamine treatment showed a decreasing trend as development progressed (compare treatment at 48 h to 96 h). Taken together, these results demonstrate a continuing requirement for Hh signaling in the growing caudal fin primordium during larval stages.

To assess whether Hh signals act directly on ACFP mesenchymal cells, we tested the cell autonomous requirement for Smoothened function in caudal fin mesenchyme by cell transplantation analysis. We used the *gfp *transgene expression as a marker to assess the ability of transplanted cells to contribute to the ACFP. We carried out cell transplantations from wild type (wt) and *smu*^-/- ^donor embryos (harboring the *gfp *transgene in 75% of the cases, see Materials and Methods) to wt and *smu*^-/- ^non-transgenic recipient embryos. All wt transgenic cells in the fin fold mesenchyme of wt recipients (200 embryos with rhodamine-dextran labeled cells in the fin fold mesenchyme) show the expected GFP signal in the proximal part of the caudal fin mesenchyme (Fig. 5E–G). In contrast, transgenic *smu*^-/- ^cells did not express GFP in a wt environment indicating a cell-autonomous requirement for *smu *(45 embryos with rhodamine-positive cells in the fin fold mesenchyme, Fig. 5H–J). As expected, transplantations of *smu*^-/- ^cells to *smu*^-/- ^recipient embryos (n = 23 embryos) did not result in rhodamine-labeled cells in the fin fold mesenchyme. However, *smu*^-/- ^cells can activate the transgene in the floor plate mimicking *shh *expression in *smu*^-/- ^embryos [30] (n = 13, Fig. 5K), and excluding the possibility that *smu*^-/- ^cells have a general defect that prevents expression of the transgene. Wt to *smu*^-/- ^transplants (n = 174 embryos) failed in all cases to contribute to the caudal fin fold mesenchyme and to express the transgene in this location. Instead, donor cells were present in the ectoderm or in the hemangiogenic mesenchyme of the tail only (Fig. 5L, M) indicating that a wild type environment with functional Smoothened is required for the correct cell fate decisions of precursor cells.

**Figure 6:**
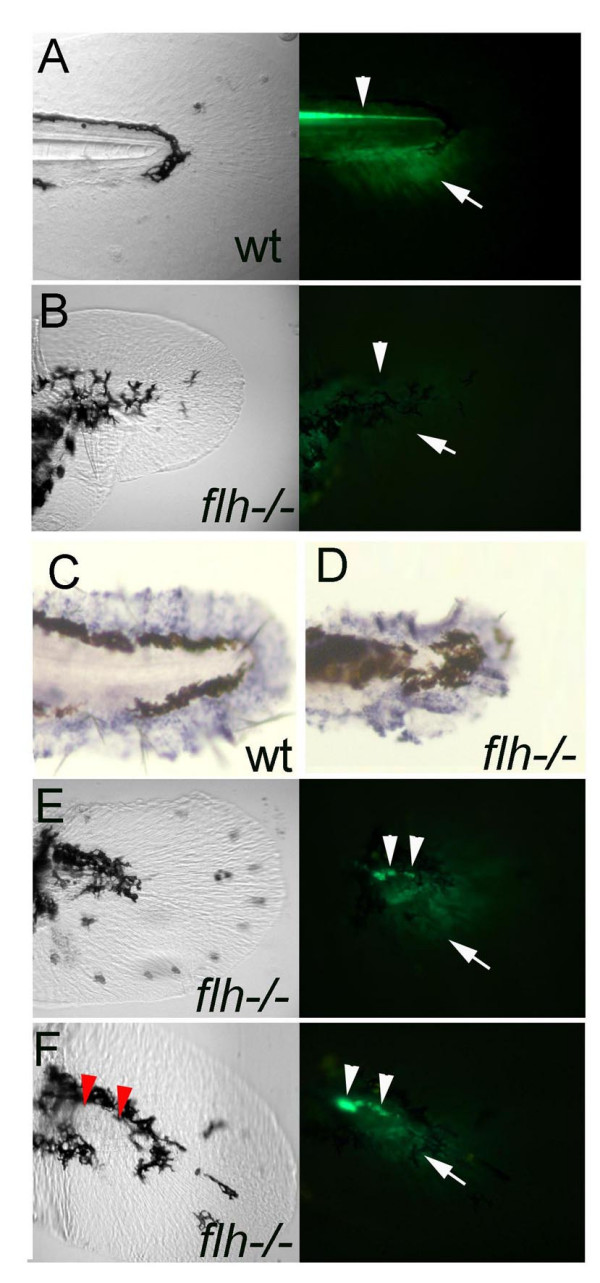
**A functional midline with expression of *shh *is required for the ACFP**. A, B, GFP expression domains including that in the floor plate indicating *shh *expression (arrowhead) and the ACFP marker (arrow) are present in wild type (A) and lost in *flh*^/- ^embryos (B). C, D, The fin fold mesenchyme as indicated by *msx-c *expression is unaffected in *flh*^-/- ^embryos. The ragged appearance of the fin fold is due to shrinkage during the whole mount in situ hybridization procedure. E, F, presence of residual floor plate cells (arrowheads) are coupled with rescue of the ACFP (arrows). Red arrowheads in F indicate the position of floor plate cells as detected in the fluorescent image. All images of caudal fin regions anterior to the left. Bright field views are shown on the left panels and fluorescence images using GFP filter on the right (A, B, E, F).

An important question is the source of the Hedgehog signals in ACFP patterning. The fin fold mesenchyme has been found to be devoid of *hh *expression, however, the near-by notochord and floor plate, the closest tissues that express *shh *are good candidates for providing such signals (Fig. 1A#150;G). To address the requirement for a functional midline in ACFP patterning, *floating head *(*flh*) mutants were utilized, which lack notochord and all or most of the posterior floor plate [36] and as a result, show no expression of *shh *in the trunk and tail [37]. At 3 dpf lack of midline *shh *activity is demonstrated in *flh*^-/- ^mutant embryos by the *shh *promoter driven GFP (arrowheads in Fig. 6A, B). The lack of functional midline was coupled with the lack or substantially reduced GFP activity in the ACFP of the fin fold mesenchyme (in 96.2% of embryos, n = 62, arrows in Fig. 6A, B) while the fin fold mesenchyme remained unaffected as indicated by *msx-C *(Fig. 6C, D). This result indicates that a functional midline is required for the patterning of the ACFP. If midline plays a role in ACFP patterning by providing Shh, it is expected that in *flh*^-/- ^mutants in which a small number of Shh expressing residual floor plate cells occasionally appear, the ACFP phenotype may be rescued. To address this possibility we have exploited the residual floor plate cells in *flh*^-/- ^mutants and compared the ACFP regions of *flh*^-/- ^embryos with or without floor plate cells (arrowheads in Fig. 6B, E, and 6F). Most of the embryos with floor plate cells (70.9%, n = 85) showed distinguishable recovery of the ACFP (arrow in Fig. 6E, F). In contrast, *flh*^-/- ^embryos without floor plate cells showed ACFP GFP recovery only in 26.9% (n = 52) of the embryos analyzed. This result indicates that functional midline cells are associated with the ACFP and *shh *expressing cells of the midline likely contribute to the rescue of the ACFP patterning. The fact, that *syu*^-/- ^mutants have both floor plate and notochord, but lack functional Shh, together with the above results in *flh*^-/- ^mutants is consistent with the suggested role of the midline in ACFP patterning to provide Shh signals.

## Discussion

In summary, gene expression analysis, cell transplantations, mutant analysis and conditional Hh inhibition experiments indicate a direct requirement for Shh signaling during early patterning of the ACFP. In contrast to the ZPA of wings, paired fins and limbs of Osteichthyes, *shh *is not expressed within the ACFP. However, several lines of evidence suggests that the ACFP is an early target of Hedgehog signals and that Shh originates from the notochord and floor plate indicating a long distance delivery mechanism functioning in the caudal fin primordium. This finding provides the earliest acting molecular mechanism uncovered that function in the caudal fin primordium of Osteichthyes and brings into focus the question of the evolutionary origin and mechanism of Hh signaling in vertebrate appendage specification.

The local expression of several Hedgehog signaling pathway components and the lack of *hedgehog *gene expression in the ACFP is consistent with a long distance Hedgehog signaling mechanism while it does not exclude the possibility that Hh signaling also acts on premigratory or migrating precursor cells. The *you *(Scube2) gene was identified as a component of the Shh pathway component acting at long distance [25,26] which is active in Shh receiving cells and shows high expression levels away from the source of Shh in the neural tube. Interestingly, the *you *gene is already expressed in the embryonic fin fold in the caudal region at 1 dpf [25] and is specifically expressed in the ACFP (this study). However, the *you *gene is redundant in the patterning of the ACFP probably due to the existence of several complementing *you *paralog (our unpublished data). Similarly to the *you *gene, *smu *is expressed at high levels away from the source of Shh in the dorsal brain [30] and dorsal neural tube as well as in the ACFP. These expression activities together with the lack of detectable *hedgehog *gene expression provide support to the notion of a Hedgehog signaling mechanism acting at a distance in the ACFP.

The data presented in this study does not explain the exact mechanism of the Hh signaling, however several studies provide parallels from other tissues of the vertebrate embryo that may be analogous to the mechanisms present in the caudal fin primordium. A long distance Hedgehog signaling mechanism could act through migrating cells that contribute to the caudal fin primordium. For example, Hh signaling acts in the migrating murine cranial neural crest cells in mouse and fish [38,39] and a potentially similar mechanism may act in the ACFP. Recent work in lamprey and dogshark suggests that the median fins are mainly of sclerotomal origin, albeit some contribution from the neural crest has also been demonstrated in these basal vertebrates [17]. Further reports suggest that trunk neural crest contributes to the caudal fin and other median fins also in Osteichthyes [15,18,40]. Trunk neural crest cells migrate from a dorsal aspect of the trunk ventrally alongside the midline, which is a known source of Hedgehogs, which may take effect on these migrating cells. Alternatively, somitic mesoderm (sclerotome) cells migrating ventrally may also contribute to the caudal fin primordium similarly to that observed in the embryonic fin fold of axolotl [41]. Hedgehog signaling could also act on somitic cells similarly to the way Shh expressed in the midline functions in patterning the muscle pioneer cells that migrate laterally to form the slow muscle [42,43]. It is interesting to note, that in our cell transplantation experiments when wild type cells were transplanted into *smu*^-/- ^mutants, these wt cells failed to migrate to where the ACFP would normally form. This result suggests that hedgehog signaling is also required for the correct migration of cells into the ACFP and this likely represent an additional non-cell autonomous function for Hedgehog signaling, prerequisite to the formation of the caudal fin primordium. The cells of the ACFP mesenchyme, however, are unlikely to be solely originating from cell migration, and local cell proliferation within the territory, where we have identified the ACFP has also been reported [44]. The process of cell migration and local cell proliferation in the ACFP is likely regulated by non-canonical Wnt signaling, as suggested by the analysis of Wnt5a function in *hoxb8a *mutant medaka fish (Oryzias latipes) [18]. It will be interesting to address the possible regulatory relationship between Hh and Wnt signaling in the caudal fin priordium.

Whether Hedgehog signaling acts on mesodermal or neural crest derived cells in the zebrafish caudal fin is yet to be determined as the origin of the ACFP cells is yet unknown and requires cell fate mapping in developing caudal fins. Due to the late developing nature of the tail primordium novel fate mapping technologies will have to be adapted to zebrafish such as the tissue specific and conditional activation of marker genes in somitic and neural crest cells of the developing tail [45].

It cannot be excluded at this point that other *hedgehog *genes such as *shhb *and *ihhb *expressed in the midline during the formation of the ACFP may also contribute to its development. The data presented here with the *syu *mutant and the fact that *shh *is the only *hh *gene continually expressed in the midline during the time of the formation for the ACFP strongly suggests that Shh is the key protein functioning in early ACFP patterning. While direct comparisons between the paired limbs and the caudal fin can not be made due to the fundamental morphological differences between these appendages, it is interesting to note that several regulators of limb buds are active in and required for ACFP development.

It is not possible to address what adult morphology is specifically affected by the loss of Shh signaling as the Shh pathway mutants as well as cyclopamine treated embryos die before markers of adult caudal fin appear and can be analyzed. In this respect it is interesting to note that Shh was previously implicated in promoting chondrogenesis in mesenchyme cells [46]. Nevertheless, several aspects of ACFP patterning are affected besides the aberrant GFP expression in Hh pathway mutants, including the aberrant expression of *bmp4 *and mis-migration of melanophores that form the gap which marks the antero-posterior position of the ACFP in wild type embryos.

Antero-posterior (A-P) asymmetry of differentiated endoskeletal morphology is a fundamental feature of gnathostome paired and unpaired fins [20]. Due to the lack of markers for the antero-posterior domains of the ACFP our experiments on Shh signaling components, could not reveal an obvious role for these molecules in the A-P patterning of the caudal fin primordium. The expression of GFP and the additional genes studied in the ACFP all express in a similar antero-posterior extent. As it is demonstrated by the time lapse analysis of the GFP expression, these marker genes do not represent obvious antero-posterior restricted expression at least at the time of the initial formation of the ACFP domain. However, our results suggest significant modifications of the functions of common molecular components in the caudal fin in comparison to paired fins/limb buds. The expression of Shh in the ZPA has previously been suggested to contribute to the morphological changes resulting in separation of the fin from the body wall in Osteichthyes [32] and has been proposed to be co-opted after the Osteichthyes/Chondrichthyes split [32,11]. Recently, contrasting data have been published [47] demonstrating that skate (*Raja erinacea*) and a shark species (*Chiloscyllum punctatum*) possesses posterior *shh *expression in pectoral and dorsal fins, suggesting, that the *shh *expression domain in appendages is an ancient property, which may have remained undetected in dogfish [32] possibly due to the secondary loss of this expression domain in dogfish. *Shh *expression, however, was also found in the dorsal fin of skate and shark. This observation raises the question, whether polarized expression of *shh *would also be an ancient character in all median fins including the caudal fin. If this was the case, the lack of detectable *shh *expression in the zebrafish caudal fin primordium would suggest secondarily loss of *shh *expression. Unfortunately, neither of the above cited publications has addressed the expression of *shh *in the caudal fin buds of chondricthyans specifically and thus this question remains to be answered.

Alternatively, the uncovered Hh signaling function of the caudal fin primordium in the zebrafish without local *shh *source may represent ancient but fundamentally different way of fin patterning mechanism from that of paired and dorsal median fins. Although the cyprinid caudal fin, like those of other teleosts, is derived in being abbreviated and having a lepidotrichial field with a symmetry plane, the fundamental architecture of the zebrafish caudal fin is ancient. Notably, the endoskeleton of the zebrafish caudal fin contains modified forms of ancestral fin radials that during early development relate spatially to the notochord in the same manner as in the earliest gnathostomes. The molecular correlates of caudal fin formation described here are concerning the primordium of endoskeletal components of the caudal fin, as marked by the proximal GFP label of the ACFP. Thus, the patterning of the ACFP may represent the primitive gnathostome condition. In this respect, it will be particularly interesting to ask whether Hh signaling pathway components including *shh *are also expressed in the caudal fin buds of basal gnathostome and jawless vertebrates. Since median fins are evolutionarily more ancient structures than paired fins with the caudal fin being the oldest skeletal appendage of chordates [1], we speculate that the patterning of early median fin primordia by Shh signaling may be an ancient mechanism that reflects the primitive state of skeletal appendage specification in vertebrates.

## Conclusion

In this study we have provided an ontogenic description of the primordium of a teleost caudal fin, which is an important morphological specialisation of fishes and as a median fin, is considered as one of the evolutionary oldest vertebrate skeletogenic appendages. Secondly, we have gathered evidence by four independent approaches: mutant data, inhibitor drug treatment, gene expression analysis and cell autonomy analysis to demonstrate that Hedgehog signaling is a regulator of caudal fin development. Thirdly, we uncovered an intriguing difference between Hh signal delivery in paired fins that contain a Shh expressing region referred to as the zone of polarizing activity (ZPA) and the caudal fin primordium of zebrafish that we show does not express Shh. This finding provides ammunition to the debate about when and where Hedgehog signaling was first utilised in appendage development during vertebrate evolution.

## Methods

The *2.2shh:gfp:ABC#15 *transgenic line was produced as described [48]. It contains a transgene which harbors the *gfp *under control of *shh *regulatory elements responsible for *shh *expression in the floor plate and notochord [49] located within the 2.2 kb upstream of the transcriptional start site of *shh *and downstream elements embedded in the *shh *introns 1 and 2. GFP expression in embryos was detected by Nikon SMZ1500 fluorescence microscope and by Leica TCSNT confocal microscope. Alcian blue staining was carried out as described [21].

### Mutant analysis

Identified heterozygous carriers of mutant alleles (*sonic you*, *syu*^*t*4^[29], *slow muscle omitted, smu*^641^[30]*floating head*, *flhN1 *[36]) were crossed with heterozygous transgenic fish. Incross of the resulting offspring from three independent parent crosses were analyzed for the presence of mutant phenotypes and associated transgene activity.

### In situ hybridization

For producing gli3 antisense probe [24] (AY377429) a 2717 bp fragment, from sequence region most diverged from the homologous gli2 mRNA, (NM_130967) was amplified by RT-PCR using total zebrafish RNA isolated from 24 h old embryos and the following primers: forward primer: AAC GGT ACA CTG ACC CAA GC, reverse primer: TAG TGC CTG GAT CCA CAC TG. The amplified gli3 fragment was cloned directly using the Dual promoter TOPO TA Cloning Kit (Invitrogen). DIG-labeled RNA antisense probe was in-vitro transcribed with T7 RNA polymerase. In situ hybridization was performed using in vitro synthesized dygoxigenin-labeled antisense probes on whole mount zebrafish embryos at stages indicated as described [50].

### RT PCR and real time RT PCR

The ACFP region was cut out from 3 days old embryos as shown in Fig. 3 using etched tungsten micro-needles (Fine Science Tools Germany) with 1 μm tip and 125 μm in diameter. The dorsal fin fold region above the ACFP was excised as a control (DFF). To obtain comparable amount of tissue from the ACFP and DFF regions 150 samples of ACFP and 30 DFF samples were collected. The collected tissue samples were used directly for cDNA synthesis with a SuperScript™ III CellsDirect cDNA Synthesis Kit (Invitrogen). Before the addition of the reverse transcriptase enzyme (RT) a small aliquot (3 μl) was taken as a non RT control. PCR amplification from *shh smu *and *gfp *cDNA was carried out using 1 μl cDNA template from ACFP and DFF samples with the following primer pairs: *shh *FP: GACGGTCACCATTTTGAAGAATC, RP: GAGTTTACTGACATCCCCAAAGG *smu *FP: GTACACGCACACGTCTCTGATTC, RP: ATTGGCCTGAAGTGTTGAATTTG, *gfp *FP: GTCAGTGGAGAGGGTGAAGG RP: TCGCCAATTGGAGTATTTTG. Real time PCR reactions were carried out on ABI Prism SDS 7000 machine (Applied Biosystems) using ready SYBR Green mix (Qiagen). The results were analyzed with the manufacturer's software. The following primer pairs were used for the amplification:*shh *FP ACTGTCTCGCCTAGCTGTGG RP CCTTCTGTCCTCCGTCCTG, *shhb *FP AGTGGAGGCAGGATTCGAC RP CTTTGATGGGTTTCCTCGTC, *ihha *FP CCGGTTTTGATTGGGTCTAC RP GCTGCAAGCTGTCCAAAGTC, *ihhb *FP AATCCAAAGGCCACGTACAC RP TCAGAGGCCAGAACCAAGTC, *dhh *FP ATACGGCCTACTTGCACAGC RP TCAGCCATTGTCACAAGTCC, *smu *FP CACGCACACGTCTCTGATTC RP TCCACCTTTCCATTCTCACAC, *gfp *FP ACAAGCAAAAGAACGGCATC RP AAAGGGCAGATTGTGTGGAC, *β-actin *FP TACAATGAGCTCCGTGTTGC RP CACAATACCAGTAGTACGACCAGA. For comparable amount of tissue samples (normalized by β-actin amplification) 1 μl cDNA from ACFP sample and 0.4 μl cDNA from DFF sample were used as template for the real time PCR. Three technical repeats were carried out for each gene analyzed as well as a no template control for each primer pair and a non rt-control for *gfp*. Whole caudal fin samples from three developmental stages (2.5, 10 and 15 dpf) were collected by cutting the tail fin approximately on the level of the anterior end of the ACFP region, using micro-fine dissecting knife (Fine Science Tools – FST). To reduce the amount of the non-cellular material in the fin samples from the late stage larvae (15 dpf) the posterior part of the fin rays were excised. In each RT PCR reaction 0.5 μl of the cDNA samples were used.

### Cyclopamine treatment

*gfp*^+/- ^transgenic zebrafish embryos were placed individually in wells of 96-well tissue culture plates and treated at the indicated stages and times with either 2% ethanol in 10% Hank's solution or with 200 μM cyclopamine (Biomol Int., No GR-334) dissolved in 2% ethanol, 10% Hanks's solution. Incubation with cyclopamine was followed by washing in 10% Hank's and embryos were incubated until the time of GFP analysis as described. Experiments were repeated independently 3 times and images from one representative experiment are shown.

### Cell transplantation

Transplant experiments were carried out essentially as described in [51] with the following modifications. Instead of biotin, 1 pl 1% fixable tetramethyl-rhodamine-dextrane 3000 MW (Molecular Probes, D-3308) was injected into 1–4 cell wt containing the *gfp *transgene and *smo*^-/- ^embryos containing the *gfp *transgene which were left to develop till 30% epiboly. (Only embryos labeled uniformly at this stage were used for the transplants.) Embryos were then transferred into a dish where they were held in pairs during and after the transplants. The transplants were carried out between shield and 60% epiboly stage. Embryos that showed labeling on the ventral side of the median finfold were sorted at 1 dpf (wt to wt, wt to *smu*^-/-^, *smu*^-/- ^to wt, *smu*^-/- ^to *smu*^-/-^) and were left to grow till 3 dpf. Embryos were then anaesthetized and mounted in low melting point agarose and a Leica TCSNT confocal microscope was used to obtain the images of rhodamine labeling and GFP expression.

## Authors' contributions

YH carried out the molecular genetic studies and participated in the transgenic analyses. ZsL carried out the cell transplantation experiments, SS carried out the in situ hybridization experiments, SW, PA and US participated in the design of the study and in the writing of the ms, FM conceived and coordinated the study, participated in mutant analyses and wrote the ms. All authors read and approved the final manuscript.

## Supplementary Material

Additional File 1 **Figure S1. The first 20 days of caudal fin ontogeny in the zebrafish as detected by GFP activity marking the caudal fin primordium and differentiating caudal fin structures**. The animation has been assembled from individual still images taken once per day from 24 hpf on lateral view of the tail region of a zebrafish embryo/larva kept at 28°C. For images the specimen has been anaesthetized temporarily and immobilized on an agar coated plate. Click here for file

Additional File 2 **Figure S2. Development of dorsal and anal median fins of the zebrafish**. Left side panels show bright field view of dorsal fin, right panels show fluorescence signals of GFP activity. A-D: Ontogeny of the dorsal fin is marked by GFP activity. First signal is observable at 14 dpf. E, G: Development of the anal fin. GFP activity is first detected at 15 dpf. The GFP territory expands and splits into domains of the endoskeletal mesenchyme (arrow in G) and the fin ray tip (arrowhead in G) similarly to the caudal fin. H-K: Proximal expression of GFP in differentiating caudal fin primordium (arrowheads in H, I) is only observed in transgenic line #15, but not in line #24 (arrowheads in J, K), while GFP activity in the distal fin rays is present in several transgenic lines containing the *shh *regulatory elements (arrows in I, K) mimicking endogenous *shh *expression of the fin rays [21]. Age of larvae is indicated in days post fertilization (d). Click here for file

Additional File 3 **Figure S3. GFP marks pectoral fin development**. A, B: GFP marks the distal tip of the fin bud from 40 hpf onwards (arrow in A). Bright field view on the left, and fluorescence in the right. B, At 5 dpf GFPexpression is restricted to dorsal half of the mesenchyme disc (arrowheads). Bright field view on the left and fluorescence on the right are shown. C, Expression of GFP is also present in the developing fin rays at 16 dpf (arrowhead). Age of larvae developing at 28°C are indicated in days post fertilization (d). Click here for file

Additional File 4 **Figure S4 Dissociation curves of the products from the RT-PCR shown on **Fig 3J. The red and blue curves represent the dissociation of the products from ACFP and DFF samples respectively. The green and purple curves show the dissociation of the non-template (primer) controls. Specific products of *shh shhb *and *ihhb *(panel A, B and D) have been detected only in the DFF samples, but not in the ACFP. The presence of *ihha *(panel C) was not detectable either in the ACFP or in the DFF samples. The presence of *gfp *and *beta-actin *(panel E and F, respectively) were detectable in both, ACFP and DDF samples. In case of *gfp*, dissociation curves of the primer controls are very similar to that of the PCR product, due to primer dimmers, however no amplification was observed on the amplification plots (data not shown). Click here for file

Additional File 5 **Figure S5: Cyclopamine treatment results in reduction of the GFP expression domain in the ACFP**. Average width of GFP expression domain measured from the bottom of notochord in μm is shown with standard deviation. Time of cyclopamine exposure from fertilization in hours (h) is indicated. Blue bars represent 2% ethanol treated controls, bars in purple represent cyclopamine treated embryos. Number of embryos analyzed is indicated at the base of the bars. Click here for file
