# Supplementary figures and images for: Hedgehog signaling patterns the outgrowth of unpaired skeletal appendages in zebrafish
Source: BMC Dev Biol. 2007 Jun 27;7:75. doi: 10.1186/1471-213X-7-75 (PMC1950712; doi:10.1186/1471-213X-7-75)

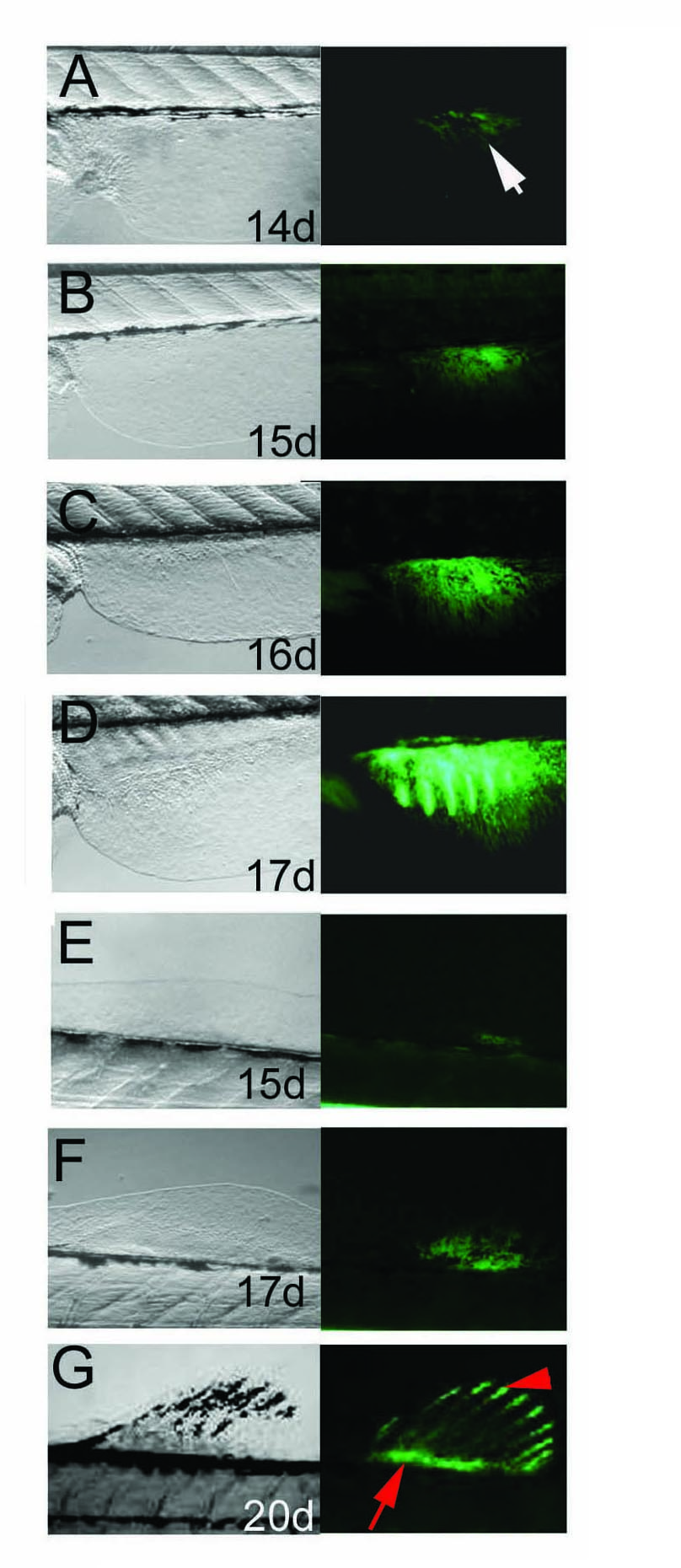

Supplement: Additional File 2 — Figure S2. Development of dorsal and anal median fins of the zebrafish. Left side panels show bright field view of dorsal fin, right panels show fluorescence signals of GFP activity. A-D: Ontogeny of the dorsal fin is marked by GFP activity. First signal is observable at 14 dpf. E, G: Development of the anal fin. GFP activity is first detected at 15 dpf. The GFP territory expands and splits into domains of the endoskeletal mesenchyme (arrow in G) and the fin ray tip (arrowhead in G) similarly to the caudal fin. H-K: Proximal expression of GFP in differentiating caudal fin primordium (arrowheads in H, I) is only observed in transgenic line #15, but not in line #24 (arrowheads in J, K), while GFP activity in the distal fin rays is present in several transgenic lines containing the shh regulatory elements (arrows in I, K) mimicking endogenous shh expression of the fin rays [21]. Age of larvae is indicated in days post fertilization (d). [file 1471-213X-7-75-S2.jpeg]

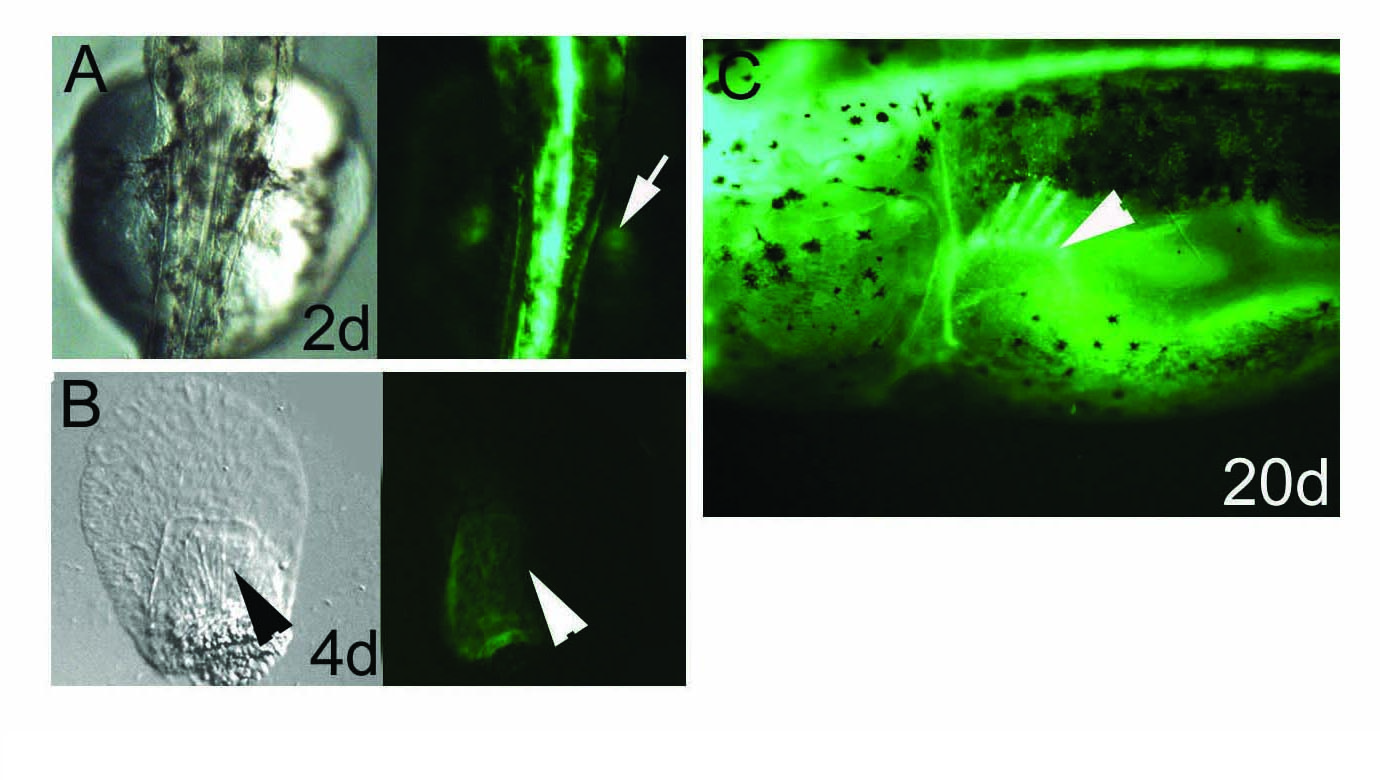

Supplement: Additional File 3 — Figure S3. GFP marks pectoral fin development. A, B: GFP marks the distal tip of the fin bud from 40 hpf onwards (arrow in A). Bright field view on the left, and fluorescence in the right. B, At 5 dpf GFPexpression is restricted to dorsal half of the mesenchyme disc (arrowheads). Bright field view on the left and fluorescence on the right are shown. C, Expression of GFP is also present in the developing fin rays at 16 dpf (arrowhead). Age of larvae developing at 28°C are indicated in days post fertilization (d). [file 1471-213X-7-75-S3.jpeg]

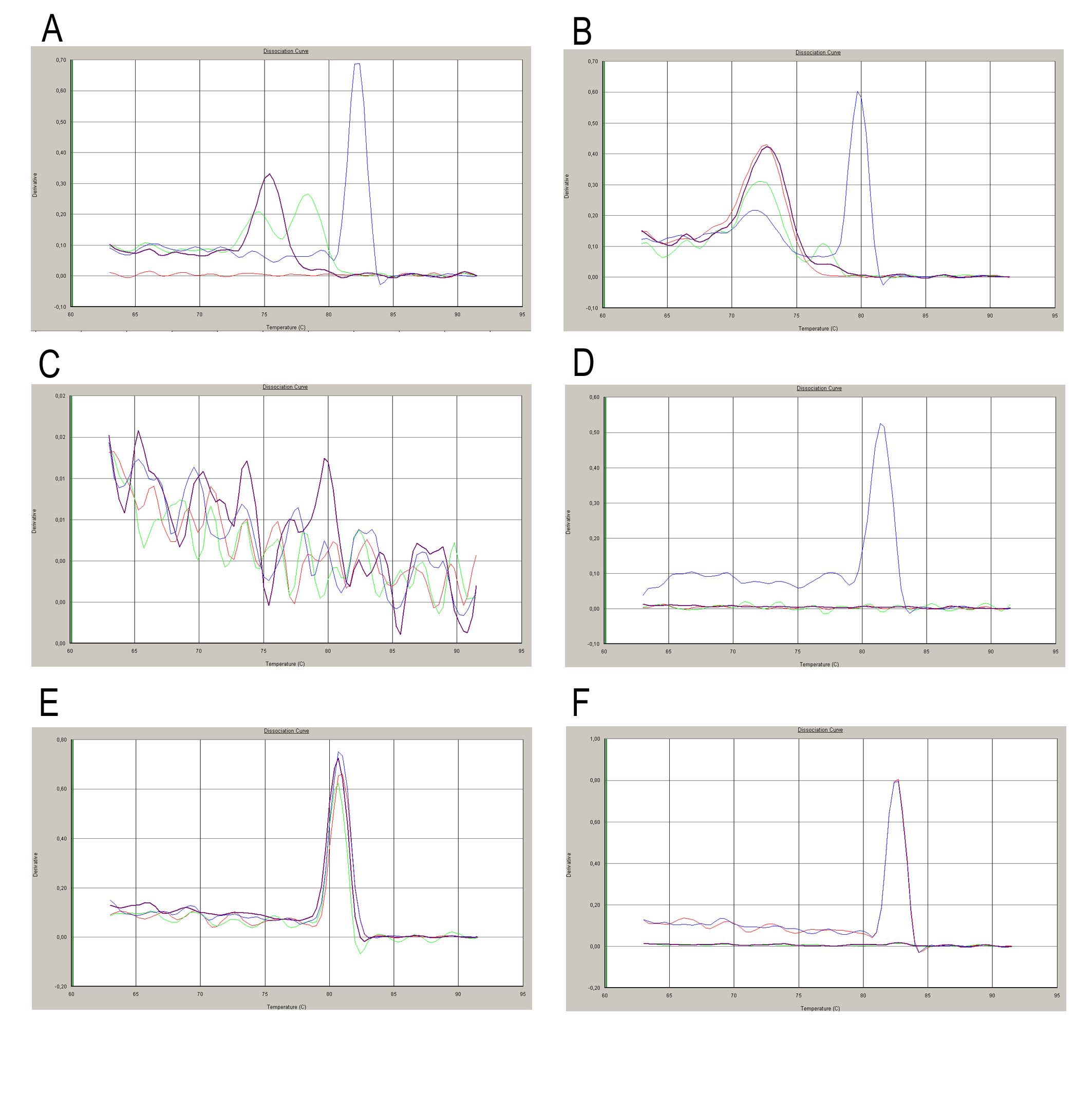

Supplement: Additional File 4 — Figure S4 Dissociation curves of the products from the RT-PCR shown on Fig 3J. The red and blue curves represent the dissociation of the products from ACFP and DFF samples respectively. The green and purple curves show the dissociation of the non-template (primer) controls. Specific products of shh shhb and ihhb (panel A, B and D) have been detected only in the DFF samples, but not in the ACFP. The presence of ihha (panel C) was not detectable either in the ACFP or in the DFF samples. The presence of gfp and beta-actin (panel E and F, respectively) were detectable in both, ACFP and DDF samples. In case of gfp, dissociation curves of the primer controls are very similar to that of the PCR product, due to primer dimmers, however no amplification was observed on the amplification plots (data not shown). [file 1471-213X-7-75-S4.jpeg]

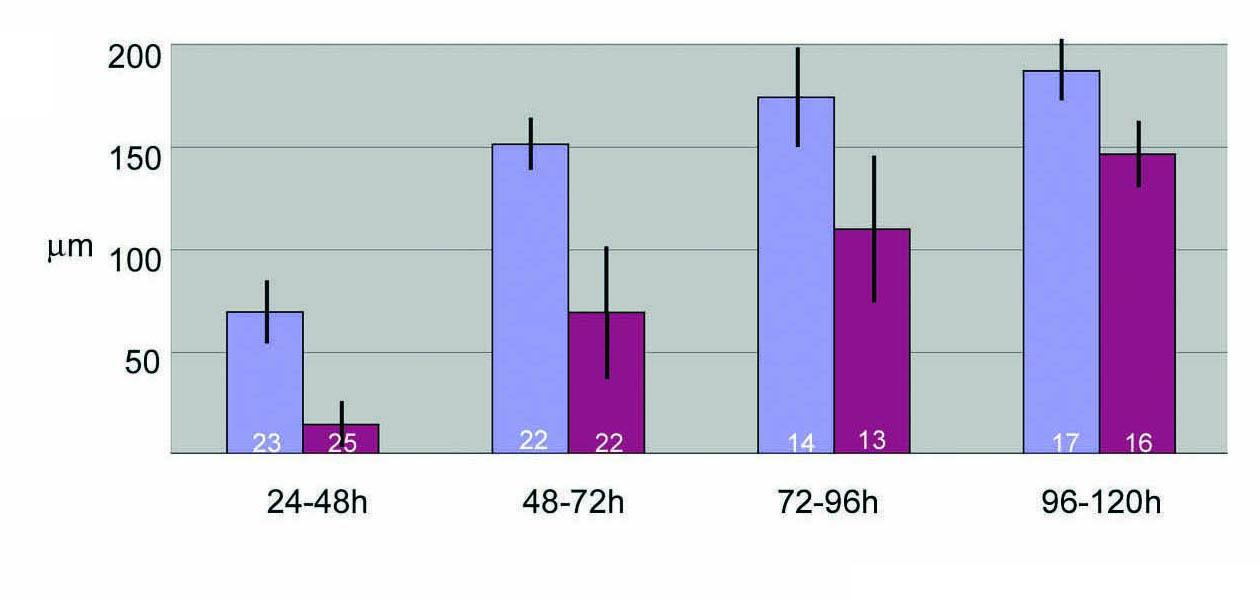

Supplement: Additional File 5 — Figure S5: Cyclopamine treatment results in reduction of the GFP expression domain in the ACFP. Average width of GFP expression domain measured from the bottom of notochord in μm is shown with standard deviation. Time of cyclopamine exposure from fertilization in hours (h) is indicated. Blue bars represent 2% ethanol treated controls, bars in purple represent cyclopamine treated embryos. Number of embryos analyzed is indicated at the base of the bars. [file 1471-213X-7-75-S5.jpeg]
